# Cross-Sectional Assessment of Nutritional Status, Dietary Intake, and Physical Activity Levels in Children (6–9 Years) in Valencia (Spain) Using Nutrimetry

**DOI:** 10.3390/nu16162649

**Published:** 2024-08-10

**Authors:** María Morales-Suárez-Varela, Isabel Peraita-Costa, Agustín Llopis-Morales, Agustín Llopis-González

**Affiliations:** 1 Research Group in Social and Nutritional Epidemiology, Pharmacoepidemiology and Public Health, Department of Preventive Medicine and Public Health, Food Sciences, Toxicology and Forensic Medicine, Faculty of Pharmacy and Food Sciences, Universitat de València, Av. Vicent Andrés Estelles s/n, 46100 Burjassot, València, Spain; isabel.peraita@uv.es (I.P.-C.); allomo3@alumni.uv.es (A.L.-M.); agustin.llopis@uv.es (A.L.-G.); 2 Biomedical Research Center in Epidemiology and Public Health Network (CIBERESP), Carlos III Health Institute, Av. Monforte de Lemos 3-5 Pabellón 11 Planta 0, 28029 Madrid, Madrid, Spain

**Keywords:** nutritional epidemiology, nutritional assessment, pediatric nutrition, sedentary behaviors

## Abstract

The aims of this research were to evaluate the current nutritional status, dietary intake, and level of physical activity and assess the need for intervention. This was a cross-sectional study with 2724 participating children aged 6–9 years old. Nutritional status was assessed using nutrimetry, dietary intake with a 3-day food-recall questionnaire and physical activity with an ad hoc questionnaire. The nutricode with the highest prevalence was healthy weight/normal stature, with 51.3% of the sample. For the BMI for age Z-score, those in the overweight/obesity category represented 37.5% of the sample, while the thinness category included 7.6%. Intake of calories, proteins, sugar, lipids, SFA, MUFA, and cholesterol were significantly higher than recommended. The thinness groups consumed a significantly higher amount of excess calories while the overweight/obesity groups had the lowest mean excess calorie intake. Children in the thinness category presented the highest rates at both ends of the spectrum for sedentary activities. This study showed the high prevalence of malnutrition in schoolchildren. The results for the risk of thinness and overweight/obesity according to individual nutrient intake should be carefully interpreted. Lifestyle is a fundamental aspect to consider when combating malnutrition, especially at the level of dietary and physical activity habits, to combine various methods of intervention to improve nutritional status.

## 1. Introduction

According to the WHO, the biggest threat to public health worldwide is malnutrition [1,2]. Malnutrition is a condition in which an insufficient supply or incorrect absorption of essential nutrients has an adverse effect on the cells, tissues, organs, and the body understood as a whole, which is manifested in the deterioration of its functioning and a negative change in the overall clinical picture, significantly affecting daily performance, intellectual, and emotional state [3,4]. Malnutrition is associated not only with reduced BMI but also with obesity. Obesity is defined as a paradoxical state of malnutrition, where excessive energy consumption is associated with a shortage of individual micronutrients [5].

Globally, malnutrition and overnutrition remain significant health issues, affecting populations across both developing and developed nations. The prevalence of childhood overweight/obesity in Spain has been a significant public health concern, with various studies indicating varying rates over time and across different regions, ranging from 20% to 45% [6,7,8,9,10,11].

Homs et al. (2023) reported that the prevalence of obesity among Spanish children and adolescents was 11.5%, with lower socioeconomic status strongly associated with higher obesity rates [12]. De Bont et al. (2022) found that the overall prevalence of obesity in Spanish children aged 2 to 17 peaked at 17.3% for girls aged 7 and 24.1% for boys aged 9 from 2005 to 2017 [10,13]. Bravo-Saquicela et al. (2022) conducted a systematic review and meta-analysis that revealed [14] that in children aged 7 to 13, the prevalence of excess body weight (overweight plus obesity) rose from 32.3% to 35.3% during 2011–2021 [14]. As of 2024, the latest data available report a childhood obesity rate in Spain of approximately 17% and a childhood excess weight rate of 37.3% for children aged 6 to 9 years [15].

Nittari et al. (2020) discussed the ongoing challenge of childhood obesity in Europe, particularly highlighting the high obesity rates in Southern European countries [16]. Moschonis et al. (2022) found that one in four children in six European countries were overweight or obese, with higher rates in lower-income countries and countries undergoing economic crises [17]. Corman et al. (2022) noted that childhood obesity has become a significant public health problem, with over 398,000 (10.6%) children aged 6–9 years being severely obese in Europe as of 2019 [18]. The data reflect ongoing public health challenges, with childhood obesity being a significant issue affecting long-term health outcomes.

Childhood obesity has both short- and long-term health consequences that can impact multiple aspects of an individual’s physical and psychological well-being [19]. Short-term effects include medical issues such as respiratory issues, chronic inflammation, orthopedic abnormalities, liver disease, diabetes, sleep disorders, and dyslipidemia; psychological issues such as low self-esteem, depression, and social isolation [20]; and decreased cognitive function, which can impact academic performance.

Long-term consequences are particularly severe, with childhood obesity leading to an increased risk of Type 2 diabetes, cardiovascular diseases, nonalcoholic fatty liver disease, metabolic syndrome, early puberty, autoimmune diseases, chronic inflammation, and premature mortality [21]. Additionally, childhood obesity has been linked to reduced quality of life due to social stigmatization and higher depression rates [22]. Obesity can also present an economic impact, as the burden associated with treating obesity-related conditions on the healthcare system and families is significant [23,24,25].

The prevalence of low BMI for age in Spanish children has been a notable concern, especially in the context of overall nutritional health. Vaquera et al. (2018) reported that 13% of girls and 14% of boys aged 5–10 years in Spain were underweight [26], and emphasized that both underweight and obesity were linked to similar characteristics of the family environment [26,27]. Cadenas-Sanchez et al. (2020) found that providing detailed reference standards for Spanish preschool children also highlighted the need for monitoring low BMI levels and the importance of early detection through anthropometric standards was emphasized [28]. Tapia-Veloz et al. (2022), which also used nutrimetry, also emphasized the need for comprehensive nutritional assessments in children, which can help identify and address issues of both underweight and overweight [29].

The concurrent presence in a population of undernutrition and overweight/obesity, along with the associated noncommunicable diseases, is known as the dual burden of malnutrition (DBM). It is seen most often in low- and middle-income countries but can also be present in certain populations of high-income countries such as Spain [30]. Diet is the main driver of the DBM [31,32], and the changes in food marketing, access, and purchasing over the past few decades that have changed traditional dietary patterns in Western countries have made it a nutrition reality [33,34,35,36].

In children, adequate caloric intake is necessary to provide the energy needed for daily activities and proper growth. However, the quality of these calories is equally crucial to ensure that children receive a balanced diet that supports overall health and development. Nutrient-dense foods provide essential vitamins and minerals that support various physiological functions, including the immune response, cognitive development, and bone health [37]. Malnutrition, whether from a deficiency or excess of nutrients, can have severe implications, such as stunted growth, impaired cognitive abilities, and an increased risk of chronic diseases such as obesity and cardiovascular issues [38,39].

Selem-Solís et al., in 2017, developed the “nutrimetry” method, which combines two accessible and easy anthropometric variables, the BMI for age Z-score (BAZ) and the height for age Z-score (HAZ), whose intention is to facilitate a joint interpretation of these two indicators and generate a more complete diagnosis of nutritional status [40,41]. This multifaceted approach provides detailed, personalized, and actionable insights into a child’s nutritional status and can help prevent and treat obesity, ultimately promoting healthier lifestyles and better long-term health outcomes for children.

In view of the above, the aims of this research were to evaluate the current nutritional status, applying nutrimetry using the HAZ and BAZ; to evaluate the dietary intake and its compliance with established -age and sex-specific recommendations; and to evaluate the level of physical activity and its compliance with established age- and sex-specific recommendations in a population of schoolchildren 6–9 years of age attending primary schools in the Valencian community (Spain). Additionally, with the results obtained, the objective was to be able to assess the need for what, if any, intervention is needed and to propose a model of dietary intake and/or physical activity modification that may improve nutritional-anthropometric status.

## 2. Methods

### 2.1. Setting and Design

This descriptive cross-sectional study was part of the Antropometria y Nutrición Infantil de Valencia (Valencian Anthropometry and Child Nutrition) or ANIVA project conducted on schoolchildren aged 6–9 years old attending primary school in the Valencian community during the 2016–2023 academic years.

This study complied with the guidelines of the World Medical Association’s Declaration of Helsinki. Prior to conducting this study, institutional permissions were obtained from the Autonomous Secretariat of Education and the University of Valencia (approval of the Ethics Committee: 2014/29630).

The first step in data collection was the presentation to the schools of a formal letter introducing the ANIVA project, which included the authorization granted by the Ministry of Education, Culture, and Sport and the University of Valencia. If a school responded and was willing to participate, an in-person meeting was arranged with the director or other staff member designated by the director and the parents’ association to more thoroughly explain the project. At the end of this introductory meeting, the school representative and parents’ association were asked if they would like to participate and, if so, the school was then considered a participating center.

Informed consent forms and information sheets were provided to the parents or legal guardians of all the eligible students, describing the objectives and methodology of the study and inviting them to participate. Parents/legal guardians were asked to sign the informed consent form and return it to the research team if they were willing to allow their children to be included in the study. Only if written informed consent was received was a child included in the study.

After parental consent was returned, the project was then explained to all the children whose parents had agreed to participate. After confirmation that all participants were informed and consenting, the parents or guardians were given an extensive data collection packet comprising several different questionnaires to complete, which aimed at collecting socioeconomic variables, variables on the students’ physical activity, and a record of the students’ dietary intake.

### 2.2. Participants

**Figure 1:**
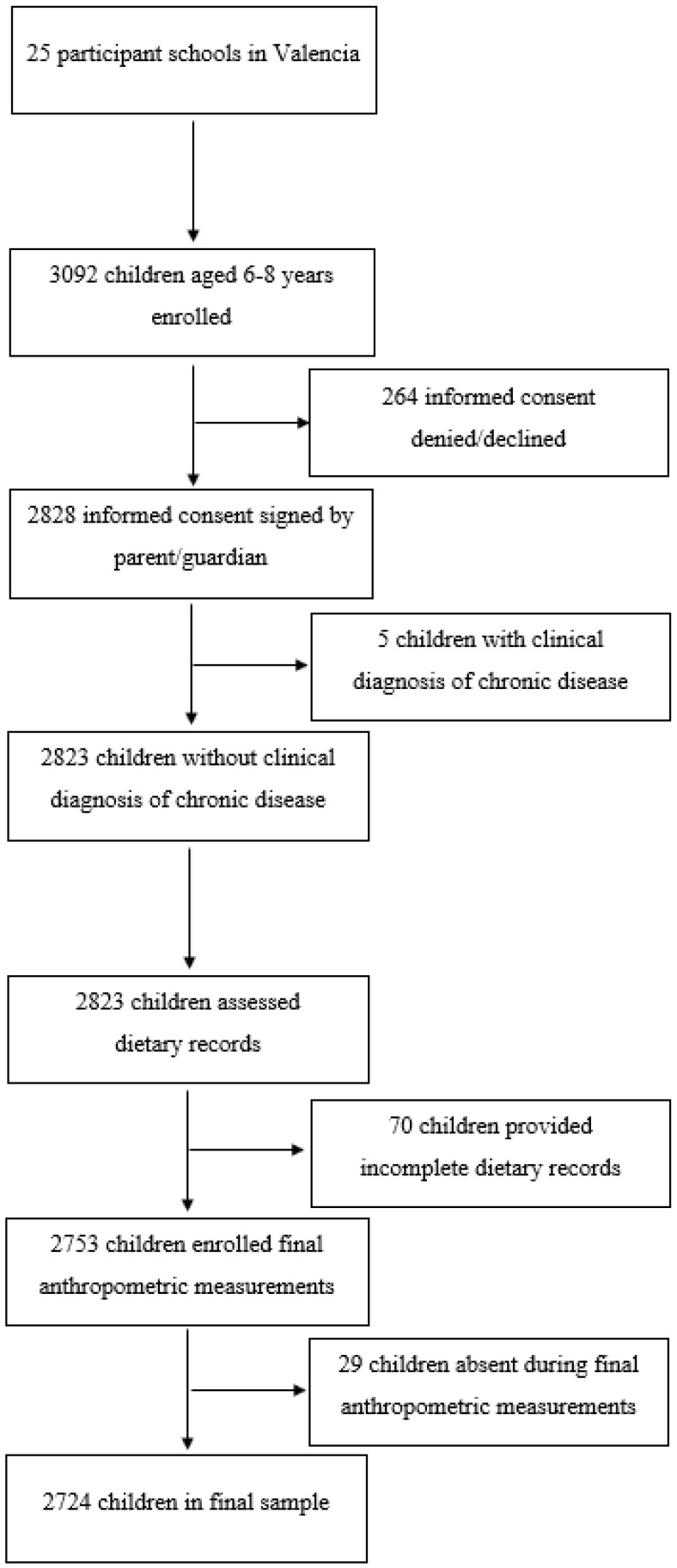
Participants’ flow diagram.

The initial sample was made up of all children aged 6 to 9 years old, of both sexes, attending the participating schools. The schools to which participation was offered were randomly selected within the Valencian community. Children were excluded if the overall data collection packet completion rate was not at least 85%, if a single questionnaire was not 85% completed, or if they were absent when the anthropometric measurements were taken. The final sample consisted of 2724 children (Figure 1), of whom 1330 were boys (48.82%) and 1394 girls (51.17%).

### 2.3. Anthropometric Measurements

All anthropometric measurements were taken with adequate supervision and in a manner that would guarantee a private and relaxed atmosphere for the participating children. In order to minimize interobserver variability, anthropometric measurements were taken following the World Health Organization (WHO) standard procedures [42,43] and in standardized conditions.

Children were barefoot and wearing light clothing, in a fasted state, had urinated, and had been sitting down and resting for 15 min when the measurements were taken. To measure weight, a Seca^®^ 861 scale (Vogel and Halke, Hamburg, Germany) was used. For height, a Seca^®^ 222 wall stadiometer (Vogel and Halke, Hamburg, Germany) was used. A Tanita^®^ Segmental-418 bioimpedance analysis system (Tanita Corp., Tokyo, Japan) was used to measure body fat percentage. Measurements were taken twice, and the mean was recorded. Body mass index (BMI, kg/m^2^) was then calculated.

A Holtain Ltd.^®^ (Crymych, UK) caliper (0.2 mm precision and a constant pressure of 10 g/mm^2^ between the valves) was used to measure skin folds. Three measurements were taken, and the mean was recorded. The triceps skin fold measurement was taken in the upper posterior region of the arm, at the midway point between the bottom of the olecranon process and the bony protrusion of the shoulder. The biceps skin fold measurement was made at the midpoint of the biceps brachii at the level of the ventral region. The suprailiac skin fold was measured in the midaxillary line, just above the iliac crest. For the abdominal skin fold measurement, using the midpoint of the navel as the initial reference point, a horizontal skin fold 3 cm to the right and 1 cm below was used.

A flexible and inextensible tape measure for measurement of perimeters + BMI (Quirumed S.L.^®^ (Paterna, Spain), model: SKBMI-64) was used to measure waist and hip circumferences. The waist measurement was taken halfway between the 10th rib and the iliac crest after a normal breath. The hip measurement was taken on the right side at the point of maximum circumference on the buttocks, placing the tape in a horizontal plane with the ground and after a normal breath. Three measurements were taken, and the mean was recorded and then used to calculate the waist–hip ratio.

Children were classified using nutrimetry, a method that makes possible the crossing of information corresponding to two different anthropometric variables in order to facilitate their joint interpretation [41]. In this study, the two anthropometric variables crossed were height for age Z-scores (HAZ) and BMI for age Z-scores (BAZ). Children were classified as thin, normal weight, or overweight/obese according to their BAZ, and as short, normal, or tall according to their HAZ using Anthro Plus^®^ computer software [44] (https://www.who.int/tools/growth-reference-data-for-5to19-years/application-tools) and the sex- and age-dependent percentile values for this specific Spanish pediatric population. The joint analysis of HAZ and BAZ yielded a combined index with 9 categories, representing different diagnoses of nutritional status, referred to as the “nutricodes” [41]. The values of prevalence for each category are shown in Table 1.

While the initial analysis was intended to be a comparison among the nine categories, the low prevalence detected in some of the categories precluded this, and it was decided that a comparative analysis would be performed between the BAZ groups for the total sample and according to sex.

### 2.4. Nutritional Intake

Nutritional intake was assessed through a questionnaire and food journal completed by the parents. In the questionnaire, the parents were asked to report food allergies or intolerances, the use of prescribed and over-the-counter medications, the use of mineral and vitamin supplements, and physical activity. The food journal was a 3-day total intake record of all the food and drinks consumed by the child and encompassed two school days and one weekend day [45,46]. Parents were asked to write down the ingredients in detail, and the serving sizes and quantities, as well as the brands of food and drinks consumed. The parents were given instructions in order to be able to correctly estimate the food and drink intake.

The levels of the reference nutritional intakes for a population allow the elaboration of dietary recommendations that ensure a balanced nutritional intake for the maintenance of good health, as well as for the development of nutritional policies to prevent deficiency and chronic diseases.

In the case of Spain, the last available update of the dietary reference intakes was carried out in 2010 by the Spanish Federation of Nutrition, Food, and Dietetics Societies (FESNAD) [45]. At the European level, the European Food Safety Authority (EFSA) has published dietary reference values between 2010 and 2017, and other countries have also updated their nutritional references in the last 10 years.

In the case of macronutrients and energy, those established by EFSA in 2017 [47] were assumed except for fats (the EFSA has no specific recommendations), which were assumed to be those established by FAO and WHO in 2010 [48].

### 2.5. Physical Activity

The current recommendations are for children and adolescents to average at least 60 min per day of moderate to vigorous intensity, mostly aerobic, physical activity, during the course of a week [49]. Vigorous intensity aerobic activities, as well as those that strengthen muscle and bone, should be performed at least 3 days a week, and the amount of time spent being sedentary, particularly the amount of recreational screen time, should be limited [49].

In this study, children were classified as sedentary/lightly active (<60 min moderate daily physical activity), moderately active (>60 min moderate daily physical activity), or very active (>60 min vigorous daily physical activity) The physical activity level factor (PAL) was calculated individually for each child, considering the child’s age, the type/intensity of daily physical activity, and the duration of daily physical activity.

### 2.6. Energy

Basal metabolic rate (BMR) and total body energy expenditure (TBEE) were calculated individually for each child considering the child’s sex, age, weight, and height. BMR was calculated using the Schofield equation (boys: 19.6 × W (kg) + 130.3 × H (cm) + 414.9; girls: 16.97 × W (kg) + 161.8 × H (cm) + 371.2) and TBEE = BMR * PAL in accordance with the recommendations of the Spanish Society of Pediatrics [50].

### 2.7. Statistical Analysis

The normality of the distribution of continuous variables was verified using both graphic (probability diagrams) and statistical (the Kolmogorov–Smirnov test) methods. All the variables were found to have a normal distribution; therefore, the parametric test was used in the analysis.

Anthropometric and nutrient intake variables are presented as the mean and standard deviation, or frequencies and percentages. The quantitative variables were analyzed using Student’s *t*-test. The Chi-square test (with Bonferroni correction) was used to compare the differences between variables. The data were analyzed using the IBM SPSS 26 Statistics software, and the criterion of statistical significance was established at *p* ≤ 0.05.

In addition, logistic regression analysis was performed to determine the risk (odds ratio (OR) with the 95% confidence interval (95%CI)) of a nutritional status of thinness or overweight/obesity depending on the adherence or nonadherence to the recommended nutrient intakes. Both the crude risk (ORc) and the risk adjusted for PAL (ORa) were calculated.

## 3. Results

Of the 2724 children included in the final sample of the study, 1330 (48.82%) were male and 1394 (51.17%) were female. The nutrimetry classifications of the sample are shown in Table 1. Around half (54.9%) of the children fall into one of the healthy weight nutricodes, while almost all (90.6%) presented a normal stature. The nutricode with the highest prevalence was that of healthy weight/normal stature, with 51.3% of the sample.

For BAZ, the second most common nutricodes were those in the overweight/obesity category, with 37.5% of the sample, while the thinness category included 7.6% of the sample. For HAZ, 8.4% of the sample fell into the tall stature group and 1.0% into the short stature group. When the sample was analyzed by sex, the distribution among the nutricodes remained very similar, with no significant differences observed between the sexes.

Table 2 presents the daily mean dietary intakes for the nutrients studied. Intake of calories, proteins, sugar, lipids, SFA, MUFA, and cholesterol were significantly higher than recommended. Water intake was lower than recommended, and carbohydrate, fiber, and PUFA intakes were within the recommendations.

In the total sample, there were significant differences in the intake of lipids, SFA, and MUFA among the BAZ categories, with those in the healthy weight group presenting the highest mean intakes. When separated by sex, this trend disappeared for boys but was still present in girls, where additional differences in PUFA and calorie intake were detected and the trend of the healthy weight group having the highest intakes continued.

Table 3 presents variables related to physical activity and sedentarism/physical inactivity. For the whole sample, differences were observed for the time the children usually watched TV on weekdays and the time the children usually spent playing on video games, computers, or the internet on weekends. These significant differences only appeared for boys when stratifying the sample by sex; in addition, a significant difference was detected in the parents’ answer to the question about children having a cell phone.

Regarding weekday TV time, children in the thinness category presented the highest rates at both ends of the spectrum: those that watch no TV and those that watch TV > 3 h/day. Meanwhile, children in the overweight/obesity group had the highest rates of weekday TV time or 1–3 h/day. The same pattern appeared for weekend time spent playing video games, or on the computer or internet. Children in the thinness category presented the highest rates at the extreme options (nothing and <3 h), and those in the overweight/obesity group had the highest rates for the option of a time of 1–3 h/day. When it came to the parent-reported cell phone ownership by the child, the lowest rates of ownership were found in the thinness group and the highest in the overweight/obesity group.

Data on energy expenditure and physical activity level are shown in Table 4. Differences were found for all the variables calculated and studied, with the only exception of PAL in girls. As expected, BMR was lowest in the thinness groups and highest in the overweight/obesity groups. The same was true for TBEE, which is related to the fact that the PAL was the same for the healthy and thinness groups, and only slightly higher in the overweight/obesity groups. The results for excess calories (difference between calorie intake and TBEE) showed that the thinness groups consumed a significantly higher amount of excess calories (<700 kcal) than the other groups and, at the same time, were the group with the lowest rate of children with <100 excess calories (6.3%). On the other side of this were the overweight/obesity groups, which had the lowest mean excess calorie intake (<300 kcal) and the highest rate of children with <100 excess calories (12.1%). When stratifying by sex, the amounts of excess calories varied significantly between the sexes, with boys consuming a higher number of calories, but the trends among the nutricode groups remained the same.

Table 4 also shows the theoretical TBEE and excess calories if PAL for each child was taken to have the recommended “active” value of 1.6 or 1.85, the value for “very active”. In both cases, the same trend observed continued, with those in the thinness groups consuming a significantly higher amount of excess of calories than the other groups and the overweight/obesity groups consuming the lowest mean excess calories. It must be noted that for girls in the overweight/obesity groups, a PAL of 1.85 would entail a caloric deficit of ~200 kcal. That was the only case in which an actual or theoretical caloric deficit was observed, but for boys in the overweight/obesity groups a PAL of 1.85 would reduce their caloric excess to within the ≤100 kcal range.

In Table 5, the crude and adjusted odds ratios for thinness and overweight/obesity according to individual nutrient intake are shown. All significant results except one appeared in the comparison between the healthy weight and overweight/obesity groups. The only one that appeared in the comparison between the healthy weight and thinness groups was the ORa in boys for SFA intake, which suggested that an intake above the recommendation protects children from thinness.

In the comparison between the healthy weight and overweight/obesity groups for the sample as a whole, consuming calories under the recommendation, consuming protein under the median, consuming sugar over the median, consuming lipids over the recommendation, consuming SFA over the recommendation, and consuming MUFA over the recommendation all had ORas that suggested a protective effect against overweight/obesity. On the other hand, consuming calories over the recommendation and consuming carbohydrates under the recommendation were risk factors for overweight/obesity.

In the comparison between the healthy weight and overweight/obesity groups for boys, consuming calories under the recommendation, consuming protein under the median, and consuming SFA over the recommendation all have ORas that suggested a protective effect against overweight/obesity. On the other hand, only consuming lipids under the recommendation is a risk factor for overweight/obesity.

In the comparison between the healthy weight and overweight/obesity groups for girls, consuming calories under the recommendation, consuming protein under the median, consuming lipids over the recommendation, consuming SFA over the recommendation, and consuming MUFA over the recommendation all have ORas that suggested a protective effect against overweight/obesity. On the other hand, consuming calories over the recommendation and consuming carbohydrates under the recommendation were risk factors for overweight/obesity.

These results related to the risk of overweight/obesity related to compliance with the nutritional intake recommendations should be carefully interpreted.

## 4. Discussion

This work studied the nutritional status of schoolchildren in the Valencian community using nutrimetry. To the authors’ knowledge, this has only been performed once previously on a much smaller sample of a Spanish pediatric population [51].

The prevalence of overweight/obesity was 37.5% and the prevalence of thinness was 7.6%. Previous studies in similar populations have reported similar results, with overweight ranging between 21.2% and 45.2% [6,7,8,9,10,11] and underweight between 0.4% and 13.3% [10,52]. The results from the 2017 Spanish National Health Survey reported the prevalence of overweight in the Valencian community at 28.2% [6]. The results of the current study would signal an increase from these previously reported, which would be in accordance with that reported by the WHO [53].

Several key factors contribute to the high childhood obesity rate in Spain, including dietary habits [33,54,55,56,57], physical inactivity [58,59,60,61,62,63], socioeconomic conditions [64,65,66,67,68], parental influence [69,70,71,72,73,74,75,76], urban living conditions [77,78,79], cultural influences [80,81,82], and lack of education and awareness [83,84].

EFSA recommends 1910.5–2110.5 kcal for boys and 1756.5–1956.5 kcal for girls per day at this age, with carbohydrates being between 45% and 60% of the total (226–301 g for boys and 209–293 g for girls), protein being 0.9 g/kg, and fat being between 20% and 35% of the total (45–78 g for boys and 41–72 g for girls) [47]. The results obtained from the analysis of mean dietary intake showed intakes outside the recommended values for most nutrients studied. Compared with previous publications from the EsNuPI Study in Spanish children aged 6–10 years old [55,85,86,87,88], intakes were higher in this sample. Other previous publications have shown that in middle childhood, diets are low in nutrient-dense foods such as fruits and vegetables, and animal-derived and fortified foods, which may lead to nutrient deficiencies that could affect growth and development [89]. Also, the practices of snacking and skipping breakfast, which are not associated with good health, are also common among children [89]. However, there are still large gaps in the available knowledge on childhood diets, especially for children in the age range studied here.

There was no clear linear association between nutritional status and mean intakes, as almost no significant differences were observed between the BAZ categories. It should be pointed out that in the few instances when significant differences were observed, the healthy weight group presented the highest mean dietary nutrient intakes and, with one exception, these intakes were all above the recommendations. The similarities observed in the intake patterns among the different nutricode groups, especially for overall caloric intake, warrant a deeper reflection on the root cause of the inadequate weight observed in 45.1% of the participating children.

These results show that apparently inadequate weight in children is not solely related to caloric intake or dietary quality. Other factors such as those discussed previously and not studied in this work may be at play here and should be further investigated.

However, the results from this study on the influence of physical activity also warrant mention, especially as this is one of the more easily modifiable factors involved. While following a healthy diet is key to ensuring proper health in children, maintaining an active lifestyle that incorporates at least 60 min of moderate/vigorous physical activity per day and limits sedentary behaviors to less than 2 h also plays a crucial role [49,90]. In this study, most participants did not meet the daily recommendations for physical activity and/or sedentary behavior. The results here, where 64.8% of the sample did not meet the physical activity recommendations, were slightly worse than those of a previous study on Spanish children, where 63.3% did not comply with the recommendations [91]. For sedentary behavior, in this work, a marked difference was observed between weekdays and weekends/holidays. While compliance with the recommendations for sedentary behaviors was the norm in the sample, at the weekend, over 75% of the sample reported behaviors of noncompliance. This was again higher than previously reported in other studies on Spanish children, where 45.6% did not comply [91]. Another study on Spanish children aged 7–10 years old showed that 58.0% of boys and 76.5% of girls did not adhere to the physical activity guidelines [92]. In that study, there was a marked difference between the sexes, which was not observed in this work.

The analysis of physical activity and energy balance carried out showed that the studied children had a lower than desired PAL and consumed, on average, an excess of around 500 kcal daily. Generally speaking, every 1 kg of weight gain needs about 7000 additional kcal; therefore, children could be gaining 1 kg every 2 weeks. The analysis also included a theoretical calculation of the energy balance after an increase in physical activity, mimicking the situation after an intervention and assuming that caloric intake remained the same, which may or may not be the case [93]. In this theoretical best-case scenario, caloric intake remained around 200 kcal above TBEE, which would still lead to weight gain, though at a slower rate than in the real scenario; it would take about 35 days to gain 1 kg.

With the results of these two analyses in mind, mean nutritional intake and physical activity/energy balance, it appears that the cause for the differences in nutritional status are still unclear, and an intervention increasing physical activity would not be sufficient to reduce the rate of overweight/obesity. This serves to highlight the known complexity of human nutrition, which is further exemplified when, other than for calories consumed, the results of the analysis used to determine the risk associated with the inadequate intake of each individual nutrient appeared to conflict with the established knowledge on nutritional intakes and health.

R. J. Stubbs et al. (1998) underscored the complexity of human feeding behavior and the interplay between energy intake and expenditure [94]. Solely for weight management, nutrient distribution is sometimes not considered to be as an important a factor, and the focus is on total energy intake. However, given these results and the known effects on health of certain nutrients, it is important to make sure that children maintain an adequate caloric intake and a balanced diet to ensure correct growth and development. The debate between counting calories and controlling the distribution of nutrient intake for weight management, which the results appear to lead us into, is nuanced, with various studies providing insights into the effectiveness of each approach.

P. Thavarajah and D. Thavarajah (2017) discussed the limitations of the conventional calorie-counting approach, emphasizing the importance of nutrient-rich foods for weight management [95]. While calorie counting provides a straightforward approach to managing energy intake, focusing on nutrient density and portion control can lead to more sustainable and holistic weight management. Nutrient-dense diets naturally limit calorie intake and improve overall health, while portion control helps individuals maintain a balanced intake without meticulous counting. In the study by Stacey J. Bell et al. (2018), participants consuming nutrient-dense meals spontaneously restricted their calorie intake without experiencing hunger, leading to significant weight loss and improved health markers [96].

Previous studies provide further insights into how nutrient intake influences the risk of obesity in children [97,98,99,100].

High-fat diets significantly contribute to obesity in children through various mechanisms, including increased adiposity, altered lipid metabolism, and inflammatory responses. Several studies have found that children consuming high-fat diets had greater adiposity and higher risks of obesity [101] and higher triglyceride to HDL cholesterol ratios [102], were linked to central precocious puberty [103], and had detrimental effects on metabolic and neurobiological systems [104]. Even maternal high-fat diets may lead to metabolic disorders and increased obesity risk in the offspring due to epigenetic changes and altered hypothalamic programming [105].

High-carbohydrate diets, particularly those high in refined sugars, contribute to obesity in children by promoting fat storage and altering metabolic health. Previous studies have found that high-glycemic carbohydrates drive obesity through their insulinogenic effects, promoting fat storage [106], while higher intakes of sugar and/or carbohydrates have been associated with higher triglyceride concentrations and lower HDL cholesterol [107] and an increased risk of obesity [108] and related metabolic disorders [109]. A rat model showed that the quality of carbohydrates, rather than the quantity, plays a critical role in the development of obesity, suggesting the importance of focusing on the type of carbohydrate in dietary guidelines [110].

High-protein diets’ impact on childhood obesity is multifaceted, with evidence suggesting both potential benefits and risks, depending on the context and duration. Studies have found that high-protein diets can improve BMI status in overweight and obese children in the short-term [111], but a high protein intake in early life increases the risk of later obesity [112]. However, several studies have shown that this is likely due to increased fat-free mass rather than fat mass [113,114,115].

Addressing both underweight and obesity in children requires multifaceted comprehensive public health strategies that encompass education, policy changes, community engagement, and individualized interventions. Effective strategies include nutritional education and awareness, policy and environmental changes, regular health screenings, community-based programs, healthcare interventions, socioeconomic support, behavioral interventions, collaboration with stakeholders, and monitoring and evaluation.

While an intervention increasing physical activity would not compensate for the amount of overeating present in the children studied, previous studies have shown the significant role of physical activity in mitigating the effects of a high nutrient intake on childhood obesity [116]. Previous studies have shown that lifestyle interventions combining PA and nutrition are effective in reducing BMI [116], blood pressure [116], low-density lipoprotein cholesterol [116], and other cardiometabolic risks such as metabolic syndrome and insulin sensitivity [117]. This improvement in insulin sensitivity and reduction in central inflammation have been associated with chronic PA and can improve neurocognitive health [118]. PA, in conjunction with healthy nutrition, is crucial for managing childhood obesity and preventing associated future health issues [119].

For this specific population, a dietary intervention would be the most effective in reducing the risk of inadequate nutritional status. It has been shown that the best nutritional interventions for preventing and combating childhood obesity appear to be those that incorporate multiple components and address dietary quality, physical activity, and behavioral aspects.

A recent systematic review highlighted that multicomponent nutritional interventions, particularly those including nutritional education, were most effective in improving dietary habits and reducing BMI and obesity [120]. Programs for improving the quality of the diet, characterized by dietary changes favoring wholegrains, fish, and vegetables over high-calorie, high-fat foods, have been shown to significantly improve reduce BMI, waist circumference, and fat mass [121]. A recent large meta-analysis indicated that while BMI changes were minimal, combined nutrition and physical activity interventions were effective in reducing obesity-related comorbidities such as metabolic syndrome and insulin resistance [117]. Overall, multicomponent interventions that integrate improvements in dietary quality, physical activity, and behavioral strategies are highlighted as the most effective.

### Strengths and Limitations

The study by Tapia-Veloz et al. (2023) illustrated nutrimetry’s effectiveness in assessing the health status of Spanish schoolchildren, highlighting its utility in identifying malnutrition and related risk factors [51]. Another study by Tapia-Veloz et al. (2022) demonstrated nutrimetry’s application in evaluating Ecuadorian schoolchildren’s nutritional status and formulating targeted educational protocols to address malnutrition [29].

Nutrimetry improves the assessment of nutritional status in different populations by providing a comprehensive and multifaceted approach. A method for assessing nutritional status that crosses two important anthropometric characteristics, it offers several benefits for understanding and managing nutrition-related health issues. It provides more specific information than individual anthropometric variables, and its use as a reference index in epidemiological studies allows us to describe and analyze the population’s distribution of malnutrition in greater detail among subgroups.

Nutrimetry allows for a more detailed and in-depth description and analysis of the population’s distribution of malnutrition by combining two suitable anthropometric indicators, that are normally used separately and interpreting them jointly. It does not require specialized equipment or software, and uses the WHO growth curves as a reference. Also, it uses numbers to convey the results, avoiding the emotional semantic load of the non-neutral language normally associated with anthropometric descriptions [40,41,122].

Certain limitations may have compromised the accuracy of the results and conclusions of this study. As for all data collected via questionnaire, there could be certain recall/recording biases as well as response bias, especially social desirability bias. The main limitations of the study may come from the fact that stigmatizing behaviors could be unreported or minimized, and healthy behaviors could be over-reported. In this situation, because parental nutritional behavior may play an important role as a model for children’s behavior, it would be interesting to know the nutritional status of the parents.

While the epidemiological data generated here are representative of the Valencian community scenario, it may not be representative of all of Spain. Future studies should be carried out in large samples in other geographic areas so that they can be representative of Spain.

All this would provide a clearer picture in order to take measures to improve the nutrition, health, diet, and lifestyle status of the Spanish pediatric population.

## 5. Conclusions

Nutrimetry can be an interesting indicator that is easy to use and interpret and is suitable for use at an epidemiological level for a more complete analysis of the nutritional status of children because it allows for the joint evaluation of HAZ and BAZ, which are typically used separately. However, more epidemiological studies are still required to fully demonstrate the importance of using nutrimetry for the comprehensive and integral nutritional evaluation of children.

This study shows the high prevalence of overweight/obesity in schoolchildren, which confirms the alarming figures worldwide and that urgent measures need to be taken to attack it from an early age and at all levels, family, school etc. However, nutrimetry classifications do not appear to be solely related to caloric intake or dietary quality; at the same time, the results show that a physical activity intervention would not be sufficient to improve anthropometric status.

Further studies are needed to elucidate the most important modifiable lifestyle risk factors for inadequate nutritional status. Lifestyle, including but not limited to dietary and physical activity habits, is a fundamental aspect to take into account in order to combine various methods that improve habits and behaviors, and, therefore, may also improve nutritional status.

## Figures and Tables

**Table 1:** Nutrimetry classification of participating children.

	BMI/Age Height/Age	Z ≤ −1 +0 Thinness	Z > −1 and Z < +1 +3 Healthy Weight	Z ≥ +1 +6 Overweight/Obesity	Total	*p*-Value ^1^
**Total**	**Z ≥ +2** **+5** **Tall stature**	14 (0.5%)	82 (3.0%)	133 (4.9%)	229 (8.4%)	0.001
	**Z > −2 and Z < +2** **+3** **Normal stature**	188 (6.9%)	1398 (51.3%)	882 (32.4%)	2468 (90.6%)	0.001
	**Z ≤ −2** **+1** **Short stature**	4 (0.1%)	16 (0.6%)	7 (0.3%)	27 (1.0%)	0.002
	**Total**	206 (7.6%)	1496 (54.9%)	1022 (37.5%)	2724 (100.0%)	0.001
	***p*-value ^2^**	0.001	0.001	0.001	0.001	
**Boys (*n* = 1330, 48.82%)**	**Z ≥ +2** **+5** **Tall stature**	7 (0.5%)	37 (2.8%)	67 (5.0%)	111 (8.3%)	0.001
	**Z > −2 and Z < +2** **+3** **Normal stature**	92 (6.9%)	663 (49.8%)	450 (33.8%)	1205 (90.6%)	0.001
	**Z ≤ −2** **+1** **Short stature**	2 (0.2%)	9 (0.7%)	3 (0.2%)	14 (1.1%)	0.009
	**Total**	101 (7.6%)	709 (53.3%)	520 (39.1%)	1330 (100.0%)	0.001
	***p*-value ^2^**	0.001	0.001	0.001	0.001	
**Girls (1394, 51.17%)**	**Z ≥ +2** **+5** **Tall stature**	7 (0.5%)	45 (3.2%)	66 (4.7%)	118 (8.5%)	0.001
	**Z > −2 and Z < +2** **+3** **Normal stature**	96 (6.9%)	735 (52.7%)	432 (31.0%)	1263 (90.6%)	0.001
	**Z ≤ −2** **+1** **Short stature**	2 (0.1%)	7 (0.5%)	4 (0.3%)	13 (0.9%)	0.111
	**Total**	105 (7.5%)	787 (56.5%)	502 (36.0%)	1394 (100.0%)	0.001
	***p*-value ^2^**	0.001	0.001	0.001	0.001	

Frequencies (% of the whole sample; total *N* = 2724; boys, *n* = 1330; girls, *n* = 1394); *p*-value < 0.05 was considered statistically significant. ^1^ *p*-value for comparison of the three BMI/age categories for each height/age category. ^2^ *p*-value for comparison of the three height/age categories for each BMI/age category.

**Table 2:** Mean daily dietary intake of the participating children by nutrimetry classification.

	Total					Boys					Girls				
	Healthy Weight (*n* = 1496, 54.9%)	Thinness (*n* = 206, 7.6%)	Overweight/ Obesity (*n* = 1022, 37.5%)	Total	*p*-Value ^4^	Healthy Weight (*n* = 709, 53.3%)	Thinness (*n* = 101, 7.6%)	Overweight/ Obesity (*n* = 520, 39.1%)	Total	*p*-Value ^4^	Healthy Weight (*n* = 787, 56.5%)	Thinness (*n* = 105, 7.5%)	Overweight/ Obesity (*n* = 502, 36.0%)	Total	*p*-Value ^4^
**Calories (kcal/day)** Boys:1910.5–2110.5 kcal/day Girls:1756.5–1956.5 kcal/day	2361.81 ± 857.42	2274.01 ± 837.31	2295.20 ± 811.82	2330.63 ± 839.55	0.090	2406.73 ± 887.57	2325.11 ± 911.94	2376.30 ± 872.77	2388.15 ± 882.48	0.631	2321.34 ± 827.81	2224.85 ± 759.82	2211.20 ± 734.92	2275.71 ± 792.82	0.041
**Proteins (g/day)** >0.9 g/kg/day	98.50 ± 36.08	97.42 ± 38.49	98.38 ± 48.53	98.41 ± 41.35	0.940	101.05 ± 38.54	100.07 ± 43.12	101.42 ± 50.31	101.12 ±43.77	0.959	96.19 ± 33.56	94.86 ± 33.44	95.24 ± 46.45	95.83 ± 38.73	0.884
**Carbohydrates (g/day)** Boys: 226–301 g/day Girls: 209–293 g/day	246.26 ± 92.04	236.47 ± 83.49	239.41 ± 105.89	243.04 ± 97.07	0.134	253.97 ± 99.87	239.58 ± 83.86	246.29 ± 88.98	249.81 ± 94.56	0.195	239.31 ± 83.84	233.47 ± 83.43	232.29 ± 120.62	236.58 ± 99.00	0.439
**Sugar (g/day)** Boys: <25 g/day Girls: <23 g/day	102.27 ± 42.10	95.42 ± 33.29	101.85 ± 112.49	101.59 ± 76.19	0.477	105.19 ± 43.58	97.44 ± 31.23	101.90 ± 41.08	103.32 ± 41.83	0.135	99.63 ± 40.56	93.47 ± 35.20	101.79 ± 155.05	99.95 ± 98.34	0.726
**Fiber (g/day)** >15 g/day	20.88 ± 11.61	19.93 ± 9.88	19.98 ± 10.92	20.47 ± 11.24	0.110	21.63 ± 12.39	20.10 ± 9.80	20.27 ± 9.59	20.98 ± 11.20	0.078	20.21 ± 10.83	19.76 ± 9.99	19.69 ± 12.14	19.99 ± 11.26	0.703
**Lipids (g/day)** Boys: 56–78 g/day Girls: 52–72 g/day	109.77 ± 42.90	105.56 ± 40.74	104.33 ± 39.93	107.40 ± 41.69	0.005	110.15 ± 38.98	107.76 ± 47.47	108.24 ± 44.10	109.20 ± 41.67	0.681	109.43 ± 46.17	103.44 ± 33.12	100.28 ± 34.68	105.68 ± 41.64	<0.001
**SFA ^1^ (g/day)** <18 g/day	37.10 ± 14.95	35.34 ± 13.20	35.16 ± 13.19	36.24 ± 14.21	0.002	37.31 ± 14.40	36.49 ± 14.72	36.55 ± 14.11	36.95 ± 14.30	0.615	36.90 ± 15.44	34.23 ± 11.52	33.73 ± 12.02	35.57 ± 14.09	<0.001
**MUFA ^2^ (g/day)** Boys: 11–33.5 g/day Girls: 10–31 g/day	47.66 ± 20.76	45.46 ± 19.89	45.70 ± 18.96	46.75 ± 20.05	0.034	47.45 ± 15.91	46.89 ± 24.91	47.34 ± 20.46	47.36 ± 18.61	0.959	47.84 ± 24.33	44.09 ± 13.40	43.99 ± 17.00	46.18 ± 21.32	0.004
**PUFA ^3^ (g/day)** Boys: <24.5 g/day Girls: <22.7 g/day	13.87 ± 6.13	13.65 ± 6.99	13.26 ± 6.86	13.62 ± 6.48	0.066	14.05 ± 6.12	13.64 ± 5.70	13.76 ± 7.77	13.90 ± 6.78	0.707	13.71 ± 6.13	13.65 ± 8.06	12.73 ± 5.74	13.36 ± 6.18	0.019
**Cholesterol (mg/day)** <300 mg/day	370.99 ± 145.36	369.80 ± 154.32	363.86 ± 189.54	368.27 ± 163.93	0.557	377.23 ± 149.63	379.23 ± 167.60	377.73 ± 225.50	377.44 ± 183.74	0.995	365.37 ± 141.26	360.73 ± 140.59	349.49 ± 142.62	359.52 ± 141.98	0.146
**Water (mL/day)** >1600 mL/day	1293.35 ± 679.11	1221.96 ± 543.40	1249.16 ± 636.18	1271.31 ± 653.78	0.132	1316.80 ± 645.23	1251.60 ± 540.47	1275.79 ± 608.62	1294.96 ± 623.27	0.397	1272.23 ± 707.95	1193.45 ± 547.27	1221.58 ± 663.00	1248.73 ± 681.10	0.298

^1^ Saturated fatty acids. ^2^ Monounsaturated fatty acids. ^3^ Polyunsaturated fatty acids. ^4^ Comparison among healthy weight, thinness, and overweight/obesity.

**Table 3:** Physical activity and sedentary behaviors of the participating children by nutrimetry classification.

	Total					Boys					Girls				
	Healthy Weight (*n* = 1496, 54.9%)	Thinness (*n* = 206, 7.6%)	Overweight/ Obesity (*n* = 1022, 37.5%)	Total	*p*-Value	Healthy Weight (*n* = 709, 53.3%)	Thinness (*n* = 101, 7.6%)	Overweight/ Obesity (*n* = 520, 39.1%)	Total	*p*-Value	Healthy Weight (*n* = 787, 56.5%)	Thinness (*n* = 105, 7.5%)	Overweight/ Obesity (*n* = 502, 36.0%)	Total	*p*-Value
**Does your child participate in extracurricular activities?**					0.766					0.951					0.928
No	162 (15.5%)	22 (15.2%)	94 (14.5%)	278 (15.1%)		66 (13.2%)	10 (13.9%)	44 (12.7%)	120 (13.1%)		96 (17.6%)	12 (16.4%)	50 (16.6%)	158 (17.2%)	
Yes	882 (84.5%)	123 (84.8%)	554 (85.5%)	1559 (84.9%)		433 (86.8%)	62 (86.1%)	303 (87.3%)	798 (86.9%)		451 (82.4%)	61 (83.6%)	251 (83.4%)	763 (82.3%)	
Total	1044 (100.0%)	145 (100.0%)	648 (100.0%)	1837 (100.0%)		499 (100.0%)	72 (100.0%)	347 (100.0%)	918 (100.0%)		547 (100.0%)	73 (100.0%)	301 (100.0%)	921 (100.0%)	
**How much physical exercise does your child do in his/her free time?**					0.575					0.238					0.575
No exercise	47 (3.2%)	9 (4.6%)	26 (2.7%)	82 (3.1%)		24 (3.5%)	4 (4.1%)	12 (2.4%)	40 (3.1%)		23 (3.0%)	5 (5.0%)	14 (2.9%)	42 (3.1%)	
<1 time per month	91 (6.3%)	12 (6.1%)	68 (7.0%)	171 (6.5%)		27 (3.9%)	3 (3.1%)	21 (4.3%)	51 (4.0%)		64 (8.4%)	9 (9.0%)	47 (9.8%)	120 (8.9%)	
1 ≥ times a month but <1 week at a time	128 (8.8%)	14 (7.1%)	84 (8.7%)	226 (8.6%)		44 (6.4%)	9 (9.3%)	32 (6.5%)	85 (6.7%)		84 (11.0%)	5 (5.0%)	52 (10.9%)	141 (10.5%)	
Weekly but <2 h	359 (24.7%)	44 (22.3%)	235 (24.2%)	638 (24.4%)		145 (21.1%)	11 (11.3%)	104 (21.1%)	260 (20.4%)		214 (28.0%)	33 (33.0%)	131 (27.3%)	378 (28.1%)	
Weekly >2 h	812 (55.9%)	113 (57.4%)	539 (55.5%)	1464 (55.9%)		440 (64.0%)	66 (68.0%)	314 (63.8%)	820 (64.3%)		372 (48.6%)	47 (47.0%)	225 (47.0%)	644 (47.9%)	
Don’t know/no answer	15 (1.0%)	5 (2.5%)	19 (2.0%)	39 (1.5%)		7 (1.0%)	4 (4.1%)	9 (1.8%)	20 (1.6%)		8 (1.0%)	1 (1.0%)	10 (2.1%)	19 (1.4%)	
Total	1452 (100.0%)	197 (100.0%)	971 (100.0%)	2620 (100.0%)		687 (100.0%)	97 (100.0%)	492 (100.0%)	1276 (100.0%)		765 (100.0%)	100 (100.0%)	479 (100.0%)	1344 (100.0%)	
**Does your child have a cell phone?**					0.118					0.015					0.542
No	1012 (97.2%)	143 (98.6%)	618 (95.1%)	1773 (96.5%)		486 (97.8%)	71 (98.6%)	327 (93.7%)	884 (96.3%)		526 (96.3%)	72 (98.6%)	291 (96.4%)	889 (96.5%)	
Yes	29 (2.8%)	2 (1.4%)	31 (4.9%)	62 (3.5%)		9 (1.8%)	1 (1.4%)	21 (6.0%)	31 (3.4%)		20 (3.7%)	1 (1.4%)	10 (3.3%)	31 (3.4%)	
Total	1041 (100.0%)	145 (100.0%)	649 (100.0%)	1835 (100.0%)		495 (100.0%)	72 (100.0%)	348 (100.0%)	915 (100.0%)		546 (100.0%)	73 (100.0%)	301 (100.0%)	920 (100.0%)	
**Do you have a cell phone?** Child’s answer					0.720					0.549					0.880
No	875 (87.2%)	125 (89.3%)	537 (86.8%)	1537 (87.2%)		419 (86.2%)	61 (91.0%)	287 (86.7%)	767 (86.8%)		456 (88.0%)	64 (87.7%)	250 (86.8%)	770 (87.6%)	
Yes	129 (12.8%)	15 (10.7%)	82 (13.2%)	226 (12.8%)		67 (13.8%)	6 (9.0%)	44 (13.3%)	117 (13.2%)		62 (12.0%)	9 (12.3%)	38 (13.2%)	109 (12.4%)	
Total	1004 (100.0%)	140 (100.0%)	619 (100.0%)	1763 (100.0%)		486 (100.0%)	67 (100.0%)	331 (100.0%)	884 (100.0%)		518 (100.0%)	73 (100.0%)	288 (100.0%)	879 (100.0%)	
**Do you use a mom/dad’s cell phone?** Child’s answer					0.112					0.349					0.297
No	410 (40.9%)	55 (39.3%)	221 (35.7%)	686 (39.0%)		194 (39.9%)	24 (35.8%)	116 (35.0%)	334 (37.8%)		216 (41.9%)	31 (42.5%)	105 (36.5%)	352 (40.1%)	
Yes	592 (59.1%)	85 (60.7%)	398 (64.3%)	1075 (61.0%)		292 (60.1%)	43 (64.2%)	215 (65.0%)	550 (62.2%)		300 (58.1%)	42 (57.5%)	183 (63.5%)	525 (59.9%)	
Total	1002 (100.0%)	140 (100.0%)	619 (100.0%)	1761 (100.0%)		486 (100.0%)	67 (100.0%)	331 (100.0%)	884 (100.0%)		516 (100.0%)	73 (100.0%)	288 (100.0%)	877 (100.0%)	
**How much time does the child usually watch TV on weekdays?**					0.005					0.009					0.347
Nothing	75 (5.2%)	13 (6.6%)	39 (4.0%)	127 (4.8%)		31 (4.5%)	5 (5.2%)	15 (3.0%)	51 (4.0%)		44 (5.8%)	8 (7.9%)	24 (5.0%)	76 (5.7%)	
<1 h	516 (35.6%)	61 (30.8%)	279 (28.8%)	856 (32.7%)		234 (34.1%)	28 (28.9%)	131 (26.6%)	393 (30.8%)		282 (36.9%)	33 (32.7%)	148 (31.0%)	463 (34.5%)	
1 h	606 (41.8%)	88 (44.4%)	456 (47.0%)	1150 (43.9%)		284 (41.3%)	41 (42.3%)	243 (49.3%)	568 (44.5%)		322 (42.1%)	47 (46.5%)	213 (44.7%)	582 (43.4%)	
2 or 3 h	232 (16.0%)	28 (14.1%)	176 (18.1%)	436 (16.6%)		124 (18.0%)	16 (16.5%)	92 (18.7%)	232 (18.2%)		108 (14.1%)	12 (11.9%)	84 (17.6%)	204 (15.2%)	
>3 h	19 (1.3%)	6 (3.0%)	14 (1.4%)	39 (1.5%)		13 (1.9%)	6 (6.2%)	8 (1.6%)	27 (2.1%)		6 (0.8%)	0 (0.0%)	6 (1.3%)	12 (0.9%)	
Don’t know/no answer	3 (0.2%)	2 (1.0%)	6 (0.6%)	11 (0.4%)		1 (0.1%)	1 (1.0%)	4 (0.8%)	6 (0.5%)		2 (0.3%)	1 (1.0%)	2 (0.4%)	5 (0.4%)	
Total	1451 (100.0%)	198 (100.0%)	970 (100.0%)	2619 (100.0%)		687 (100.0%)	97 (100.0%)	493 (100.0%)	1277 (100.0%)		764 (100.0%)	101 (100.0%)	477 (100.0%)	1342 (100.0%)	
**How long does the child usually watch TV on the weekend?**					0.474					0.070					0.072
Nothing	18 (1.2%)	4 (2.0%)	11 (1.1%)	33 (1.3%)		4 (0.6%)	2 (2.1%)	3 (0.6%)	9 (0.7%)		14 (1.8%)	2 (2.0%)	8 (1.7%)	24 (1.8%)	
<1 h	67 (4.6%)	11 (5.6%)	39 (4.0%)	117 (4.5%)		35 (5.1%)	6 (6.2%)	15 (3.0%)	56 (4.4%)		32 (4.2%)	5 (5.0%)	24 (5.0%)	61 (4.6%)	
1 h	254 (17.6%)	30 (15.2%)	156 (16.1%)	440 (16.8%)		104 (15.2%)	21 (21.6%)	82 (16.6%)	207 (16.2%)		150 (19.7%)	9 (8.9%)	74 (15.5%)	233 (17.4%)	
2 or 3 h	784 (54.2%)	110 (55.6%)	534 (55.1%)	1428 (54.6%)		370 (54.0%)	39 (40.2%)	267 (54.2%)	676 (53.0%)		414 (54.4%)	71 (70.3%)	267 (56.0%)	752 (56.2%)	
>3 h	312 (21.6%)	38 (19.2%)	219 (22.6%)	569 (21.8%)		167 (24.4%)	26 (26.8%)	122 (24.7%)	315 (24.7%)		145 (19.1%)	12 (11.9%)	97 (20.3%)	254 (19.0%)	
Don’t know/no answer	11 (0.8%)	5 (2.5%)	11 (1.1%)	27 (1.0%)		5 (0.7%)	3 (3.1%)	4 (0.8%)	12 (0.9%)		6 (0.8%)	2 (2.0%)	7 (1.5%)	15 (1.1%)	
Total	1446 (100.0%)	198 (100.0%)	970 (100.0%)	2614 (100.0%)		685 (100.0%)	97 (100.0%)	493 (100.0%)	1275 (100.0%)		761 (100.0%)	101 (100.0%)	477 (100.0%)	1339 (100.0%)	
**How much time does the child usually spend playing on video games, computer, or internet on weekdays?**					0.555					0.167					0.934
Nothing	690 (47.6%)	100 (50.8%)	437 (45.1%)	1227 (46.9%)		320 (46.6%)	52 (53.6%)	206 (41.9%)	578 (45.3%)		370 (48.6%)	48 (48.0%)	231 (48.3%)	649 (48.4%)	
<1 h	523 (36.1%)	62 (31.5%)	349 (36.0%)	934 (35.7%)		240 (34.9%)	26 (26.8%)	177 (36.0%)	443 (34.7%)		273 (37.1%)	36 (36.0%)	172 (36.0%)	491 (36.6%)	
1 h	193 (13.3%)	27 (13.7%)	148 (15.3%)	368 (14.1%)		99 (14.4%)	12 (12.4%)	88 (17.9%)	199 (15.6%)		94 (12.3%)	15 (15.0%)	60 (12.6%)	169 (12.6%)	
2 or 3 h	32 (2.2%)	6 (3.0%)	23 (2.4%)	61 (2.3%)		22 (3.2%)	6 (6.2%)	14 (2.8%)	42 (3.3%)		10 (1.3%)	0 (0.0%)	9 (1.9%)	19 (1.4%)	
>3 h	6 (0.4%)	0 (0.0%)	5 (0.5%)	11 (0.4%)		5 (0.7%)	0 (0.0%)	4 (0.8%)	9 (0.7%)		1 (0.1%)	0 (0.0%)	1 (0.2%)	2 (0.1%)	
Don’t know/no answer	5 (0.3%)	2 (1.0%)	8 (0.8%)	15 (0.6%)		1 (0.1%)	1 (1.0%)	3 (0.6%)	5 (0.4%)		4 (0.5%)	1 (1.0%)	5 (1.0%)	10 (0.7%)	
Total	1449 (100.0%)	197 (100.0%)	970 (100.0%)	2616 (100.0%)		687 (100.0%)	97 (100.0%)	492 (100.0%)	1276 (100.0%)		762 (100.0%)	100 (100.0%)	478 (100.0%)	1340 (100.0%)	
**How much time does the child usually spend playing on video games, computer, or internet on weekends?**					0.041					0.006					0.433
Nothing	168 (11.6%)	37 (18.7%)	96 (9.9%)	301 (11.5%)		58 (8.5%)	17 (17.5%)	27 (5.5%)	102 (8.0%)		110 (14.5%)	20 (19.8%)	69 (14.5%)	199 (14.9%)	
<1 h	359 (24.8%)	37 (18.7%)	214 (22.1%)	610 (23.3%)		119 (17.3%)	14 (14.4%)	79 (16.0%)	212 (16.6%)		240 (31.5%)	23 (22.8%)	135 (28.3%)	398 (29.7%)	
1 h	429 (29.6%)	60 (30.3%)	301 (31.0%)	790 (30.2%)		210 (30.6%)	27 (27.8%)	147 (29.8%)	384 (30.1%)		219 (28.8%)	33 (32.7%)	154 (32.3%)	406 (30.3%)	
2 or 3 h	402 (27.8%)	49 (24.7%)	287 (29.6%)	738 (28.2%)		243 (35.4%)	25 (25.8%)	191 (38.7%)	459 (36.0%)		159 (20.9%)	24 (23.8%)	96 (20.1%)	279 (20.8%)	
>3 h	75 (5.2%)	12 (6.1%)	62 (6.4%)	149 (5.7%)		51 (7.4%)	12 (12.4%)	45 (9.1%)	108 (8.5%)		24 (3.2%)	0 (0.0%)	17 (3.6%)	41 (3.1%)	
Don’t know/no answer	14 (1.0%)	3 (1.5%)	10 (1.0%)	27 (1.0%)		5 (0.7%)	2 (2.1%)	4 (0.8%)	11 (0.9%)		9 (1.2%)	1 (1.0%)	6 (1.3%)	16 (1.2%)	
Total	1447 (100.0%)	198 (100.0%)	970 (100.0%)	2615 (100.0%)		686 (100.0%)	97 (100.0%)	493 (100.0%)	1276 (100.0%)		761 (100.0%)	101 (100.0%)	477 (100.0%)	1339 (100.0%)	

**Table 4:** Caloric requirements and recommendations of the participating children by nutrimetry classification.

	Total					Boys					Girls				
	Healthy Weight (*n* = 1496, 54.9%)	Thinness (*n* = 206, 7.6%)	Overweight/ Obesity (*n* = 1022, 37.5%)	Total	*p*-Value	Healthy Weight (*n* = 709, 53.3%)	Thinness (*n* = 101, 7.6%)	Overweight/ Obesity (*n* = 520, 39.1%)	Total	*p*-Value	Healthy Weight (*n* = 787, 56.5%)	Thinness (*n* = 105, 7.5%)	Overweight/ Obesity (*n* = 502, 36.0%)	Total	*p*-Value
**Basal metabolic rate (BMR)**	1089.83 ± 91.40	1002.38 ± 76.38	1287.10 ± 550.14	1157.23 ± 359.37	<0.001	1088.78 ± 88.39	997.31 ± 77.69	1270.51 ± 153.63	1152.89 ± 152.57	<0.001	1090.77 ± 94.08	1007.25 ± 75.15	1304.29 ± 769.26	1161.37 ± 479.80	<0.001
**Physical activity level (PAL)**	1.54 ± 0.10	1.54 ± 0.10	1.56 ± 0.09	1.55 ± 0.10	<0.001	1.54 ± 0.09	1.54 ± 0.09	1.56 ± 0.09	1.55 ± 0.09	0.005	1.54 ± 0.10	1.54 ± 0.10	1.55 ± 0.10	1.54 ± 0.10	0.114
**PAL ≥ 1.60**	527 (35.2%)	72 (35.0%)	444 (43.4%)	1043 (38.3%)	<0.001	249 (35.1%)	32 (31.7%)	233 (44.8%)	514 (38.6%)	<0.001	278 (35.3%)	40 (38.1%)	211 (42.0%)	529 (37.9%)	0.053
**Total body energy expenditure (TBEE)**	1681.00 ± 197.26	1547.51 ± 177.38	2004.96 ± 869.68	179.45 ± 579.34	<0.001	1682.81 ± 190.90	1541.56 ± 173.68	1985.29 ± 286.91	1790.35 ± 282.09	<0.001	1679.37 ± 202.92	1553.24 ± 181.52	2025.34 ± 1206.34	1794.46 ± 761.69	<0.001
**Calorie intake**	2361.81 ± 857.42	2274.01 ± 837.31	2295.20 ± 811.82	2330.63 ± 839.55	<0.001	2406.73 ± 887.57	2325.11 ± 911.94	2376.30 ± 872.77	2388.15 ± 882.48	<0.001	2321.34 ± 827.81	2224.85 ± 759.82	2211.20 ± 734.92	2275.71 ± 792.82	<0.001
**Δ Calorie intake-TBEE**	680.81 ± 872.43	726.50 ± 855.03	290.24 ± 1190.98	537.73 ± 1020.64	<0.001	723.92 ± 890.38	783.56 ± 912.95	391.01 ± 900.58	598.29 ± 910.83	<0.001	641.97 ± 854.64	671.62 ± 795.89	185.86 ± 1424.44	479.95 ± 1112.63	<0.001
**Δ Calorie intake-TBEE ≤ 100 kcal**	130 (8.7%)	13 (6.3%)	124 (12.1%)	267 (9.8%)	<0.001	48 (6.8%)	9 (8.9%)	60 (11.5%)	117 (8.8%)	<0.001	82 (10.4%)	4 (3.8%)	64 (12.7%)	150 (10.8%)	<0.001
**TBEE if PAL = 1.60**	1743.72 ± 146.24	1603.80 ± 122.21	2059.35 ± 880.22	1851.56 ± 574.99	<0.001	1742.05 ± 141.42	1595.70 ± 124.30	2032.81 ± 245.81	1844.62 ± 244.11	<0.001	1745.23 ± 150.53	1611.59 ± 120.24	2086.85 ± 1230.80	1858.18 ± 767.68	<0.001
**Δ Calorie intake-TBEE if PAL = 1.60**	618.09 ± 859.82	670.21 ± 839.58	235.84 ± 1200.89	478.62 ± 1017.49	<0.001	664.69 ± 883.70	729.42 ± 903.01	343.49 ± 890.74	544.02 ± 901.85	<0.001	576.11 ± 863.07	613.25 ± 773.77	124.34 ± 1446.56	416.22 ± 1113.46	<0.001
**TBEE if PAL = 1.85**	2016.17 ± 169.09	1854.39 ± 141.30	2381.13 ± 1017.75	2140.86 ± 664.83	<0.001	2014.24 ± 163.51	1845.02 ± 143.72	2350.43 ± 284.21	2132.83 ± 282.25	<0.001	2017.92 ± 174.05	1863.41 ± 139.02	2412.92 ± 1423.12	2148.53 ± 887.63	<0.001
**Δ Calorie intake-TBEE if PAL = 1.85**	345.63 ± 862.44	419.61 ± 841.54	−85.92 ± 1305.55	189.31 ± 1070.82	<0.001	392.49 ± 885.13	480.09 ± 903.14	25.86 ± 899.60	255.80 ± 910.64	<0.001	303.42 ± 839.80	361.44 ± 777.61	−201.72 ± 1615.18	125.88 ± 1200.84	<0.001

TBEE = BMR * PAL.

**Table 5:** Compliance with mean daily dietary intake recommendations and associated risk of the participating children by nutrimetry classification.

	Total							Boys							Girls						
	Healthy Weight (*n* = 1496, 54.9%)	Thinness (*n* = 206, 7.6%)	ORc	ORa	Overweight/ Obesity (*n* = 1022, 37.5%)	ORc	ORa	Healthy Weight (*n* = 709, 53.3%)	Thinness (*n* = 101, 7.6%)	ORc	ORa	Overweight/ Obesity (*n* = 520, 39.1%)	ORc	ORa	Healthy Weight (*n* = 787, 56.5%)	Thinness (*n* = 105, 7.5%)	ORc	ORa	Overweight/ Obesity (*n* = 502, 36.0%)	ORc	ORa
**Calories**																					
Boys: 1910.5–2110.5 kcal/day Girls: 1756.5–1956.5 kcal/day	89 (9.4%)	9 (7.0%)	Ref.	Ref.	92 (13.6%)	Ref.	Ref.	29 (7.0%)	5 (9.6%)	Ref.	Ref.	41 (13.5%)	Ref.	Ref.	60 (11.4%)	4 (5.3%)	Ref.	Ref.	51 (13.7%)	Ref.	Ref.
Boys: <1910.5 kcal/day Girls: <1756.5 kcal/day	75 (8.0%)	7 (5.5%)	1.42 (0.69–2.90)	1.42 (0.69–2.92)	212 (31.4%)	0.46 (0.34–0.63)	0.46 (0.34–0.64)	29 (7.0%)	1 (1.9%)	0.75 (0.28–2.03)	0.69 (0.25–1.93)	71 (23.4%)	0.38 (0.23–0.63)	0.39 (0.23–0.65)	46 (8.7%)	6 (7.9%)	2.35 (0.83–6.67)	2.40 (0.84–6.85)	141 (37.9%)	0.50 (0.33–0.76)	0.49 (0.32–0.75)
Boys: >2110.5 kcal/day Girls: >1956.5 kcal/day	779 (82.6%)	112 (87.5%)	0.92 (0.33–2.60)	0.92 (0.33–2.60)	372 (55.0%)	2.73 (1.85–4.05)	2.73 (1.85–4.05)	357 (86.0%)	46 (88.5%)	0.20 (0.02–1.82)	0.20 (0.02–1.78)	192 (63.2%)	1.73 (0.91–3.29)	1.73 (0.91–3.29)	422 (79.9%)	66 (86.8%)	1.96 (0.52–7.34)	1.93 (0.52–7.27)	180 (48.4%)	3.61 (2.19–5.95)	3.63 (2.20–5.99)
Proteins *																					
>0.9 g/kg	864 (57.8%)	107 (51.9%)	Ref.	Ref.	734 (71.8%)	Ref.	Ref.	425 (59.9%)	52 (51.5%)	Ref.	Ref.	373 (71.7%)	Ref.	Ref.	439 (55.8%)	55 (52.4%)	Ref.	Ref.	361 (71.9%)	Ref.	Ref.
<0.9 g/kg	632 (42.2%)	99 (48.1%)	1.27 (0.95–1.69)	1.13 (0.78–1.65)	288 (28.2%)	0.54 (0.45–0.63)	0.57 (0.46–0.70)	284 (40.1%)	49 (48.5%)	1.41 (0.93–2.14)	1.00 (0.55–1.82)	147 (28.3%)	0.59 (0.46–0.75)	0.67 (0.48–0.92)	348 (44.2%)	50 (47.6%)	1.15 (0.76–1.73)	1.22 (0.75–1.99)	141 (28.1%)	0.49 (0.39–0.63)	0.49 (0.37–0.66)
**Carbohydrates**																					
Boys: 226–301 g/day Girls: 209–293 g/day	728 (49.3%)	99 (48.1%)	Ref.	Ref.	458 (45.1%)	Ref.	Ref.	325 (46.4%)	40 (39.6%)	Ref.	Ref.	227 (43.9%)	Ref.	Ref.	403 (51.9%)	59 (56.2%)	Ref.	Ref.	231 (46.3%)	Ref.	Ref.
Boys: <226 g/day Girls: <209 g/day	581 (39.3%)	91 (44.2%)	1.15 (0.85–1.56)	1.19 (0.81–1.74)	459 (45.2%)	1.26 (1.06–1.49)	1.23 (1.00–1.51)	293 (41.8%)	51 (50.5%)	1.41 (0.91–2.20)	1.72 (0.93–3.16)	235 (45.5%)	1.15 (0.90–1.46)	1.25 (0.91–1.70)	288 (37.1%)	40 (38.1%)	0.95 (0.62–1.46)	0.94 (0.57–1.54)	224 (44.9%)	1.36 (1.07–1.72)	1.21 (0.92–1.60)
Boys: >301 g/day Girls: >293 g/day	169 (11.4%)	16 (7.8%)	0.70 (0.40–1.21)	0.52 (0.22–1.24)	99 (9.7%)	0.93 (0.71–1.23)	0.93 (0.64–1.36)	83 (11.8%)	10 (9.9%)	0.98 (0.47–2.04)	0.82 (0.23–2.92)	55 (10.6%)	0.95 (0.65–1.39)	1.19 (0.70–2.04)	86 (11.1%)	6 (5.7%)	0.48 (0.20–1.14)	0.38 (0.11–1.28)	44 (8.8%)	0.89 (0.60–1.33)	0.73 (0.43–1.25)
Sugar *																					
Boys: <25 g/day Girls: <23 g/day	716 (48.0%)	110 (53.7%)	Ref.	Ref.	532 (52.2%)	Ref.	Ref.	343 (48.5%)	52 (52.0%)	Ref.	Ref.	267 (51.4%)	Ref.	Ref.	373 (47.5%)	58 (55.2%)	Ref.	Ref.	265 (52.9%)	Ref.	Ref.
Boys: >25 g/day Girls: >23 g/day	776 (52.0%)	95 (46.3%)	0.80 (0.60–1.07)	0.83 (0.57–1.20)	488 (47.8%)	0.85 (0.72–0.99)	0.80 (0.66–0.98)	364 (51.5%)	48 (48.0%)	0.87 (0.57–1.32)	1.06 (0.59–1.89)	252 (48.6%)	0.89 (0.71–1.12)	0.79 (0.59–1.06)	412 (52.5%)	47 (44.8%)	0.73 (0.49–1.11)	0.70 (0.43–1.14)	236 (47.1%)	0.81 (0.64–1.01)	0.80 (0.62–1.05)
**Fiber**																					
>15 g/day	234 (15.7%)	33 (16.1%)	Ref.	Ref.	136 (13.4%)	Ref.	Ref.	127 (18.0%)	17 (17.0%)	Ref.	Ref.	75 (14.5%)	Ref.	Ref.	107 (13.6%)	16 (15.2%)	Ref.	Ref.	61 (12.2%)	Ref.	Ref.
<15 g/day	1257 (84.3%)	172 (83.9%)	0.97 (0.65–1.44)	1.02 (0.58–1.80)	882 (86.6%)	1.21 (0.96–1.52)	1.19 (0.87–1.62)	580 (82.0%)	83 (83.0%)	1.07 (0.61–1.86)	1.05 (0.45–2.44)	442 (85.5%)	1.29 (0.95–1.76)	1.03 (0.67–1.60)	677 (86.4%)	89 (84.8%)	0.88 (0.50–1.55)	0.99 (0.47–2.09)	440 (87.8%)	1.14 (0.81–1.60)	1.39 (0.88–2.20)
**Lipids**																					
Boys: 56–78 g/day Girls: 52–72 g/day	136 (9.1%)	22 (10.7%)	Ref.	Ref.	129 (12.7%)	Ref.	Ref.	74 (10.5%)	12 (12.0%)	Ref.	Ref.	66 (12.7%)	Ref.	Ref.	62 (7.9%)	10 (9.5%)	Ref.	Ref.	63 (12.6%)	Ref.	Ref.
Boys: <56 g/day Girls: <52 g/day	11 (0.7%)	2 (1.0%)	1.12 (0.23–5.45)	0.74 (0.09–6.32)	21 (2.1%)	2.01 (0.93–4.34)	2.16 (0.89–5.26)	6 (0.8%)	1 (1.0%)	1.03 (0.11–9.31)	-	13 (2.5%)	2.43 (0.87–6.76)	4.08 (1.07–15.50)	5 (0.6%)	1 (1.0%)	1.24 (0.13–11.75)	1.06 (0.11–10.38)	8 (1.6%)	1.58 (0.49–5.08)	1.10 (0.31–3.90)
Boys: >78 g/day Girls: >72 g/day	1347 (90.2%)	181 (88.3%)	0.83 (0.52–1.34)	0.79 (0.45–1.39)	869 (85.3%)	0.68 (0.53–0.88)	0.66 (0.49–0.89)	627 (88.7%)	87 (87.0%)	0.86 (0.45–1.64)	0.82 (0.36–1.83)	440 (84.8%)	0.79 (0.55–1.12)	0.77 (0.51–1.18)	720 (91.5%)	94 (89.5%)	0.81 (0.40–1.63)	0.73 (0.33–1.63)	429 (85.8%)	0.59 (0.41–0.85)	0.55 (0.36–0.85)
**SFA ^1^**																					
<18 g/day	7 (0.5%)	2 (1.0%)	Ref.	Ref.	16 (1.6%)	Ref.	Ref.	5 (0.7%)	2 (2.0%)	Ref.	Ref.	8 (1.5%)	Ref.	Ref.	2 (0.3%)	0 (0.0%)	Ref.	Ref.	8 (1.6%)	Ref.	Ref.
>18 g/day	1489 (99.5%)	204 (99.0%)	0.48 (0.10–2.32)	0.27 (0.05–1.51)	1004 (98.4%)	0.30 (0.12–0.72)	0.20 (0.06–0.60)	704 (99.3%)	99 (98.0%)	0.35 (0.07–1.84)	0.13 (0.02–0.91)	510 (98.5%)	0.45 (0.15–1.39)	0.19 (0.04–0.90)	785 (99.7%)	105 (100.0%)	-	-	494 (98.4%)	0.16 (0.03–0.74)	0.20 (0.04–1.02)
**MUFA ^2^**																					
Boys: 11–33.5 g/day Girls: 10–31 g/day	185 (12.4%)	32 (15.5%)	Ref.	Ref.	164 (16.2%)	Ref.	Ref.	89 (12.6%)	18 (17.8%)	Ref.	Ref.	83 (16.2%)	Ref.	Ref.	96 (12.2%)	14 813.3%)	Ref.	Ref.	81 (16.2%)	Ref.	Ref.
Boys: <11 g/day Girls: <10 g/day	0	0	-	-	0	-	-	0	0	-		0	-	-	0	0	-	-	0	-	-
Boys: >33.5 g/day Girls: >31 g/day	1306 (87.6%)	174 (84.5%)	0.77 (0.51–1.16)	0.83 (0.50–1.37)	848 (83.8%)	0.73 (0.58–0.92)	0.73 (0.55–0.95)	618 (87.4%)	83 (82.2%)	0.66 (0.38–1.16)	0.77 (0.37–1.61)	430 (83.8%)	0.75 (0.54–1.03)	0.72 (0.49–1.06)	688 (87.8%)	91 (86.7%)	0.91 (0.50–1.66)	0.86 (0.43–1.72)	418 (83.8%)	0.72 (0.52–0.99)	0.74 (0.50–1.08)
**PUFA ^3^**																					
Boys: <24.5 g/day Girls: <22.7 g/day	1422 (95.1%)	195 (94.7%)	Ref.	Ref.	982 (96.1%)	Ref.	Ref.	676 (95.3%)	96 (95.0%)	Ref.	Ref.	499 (96.0%)	Ref.	Ref.	746 (94.8%)	99 (94.3%)	Ref.	Ref.	483 (96.2%)	Ref.	Ref.
Boys: >24.5 g/day Girls: >22.7 g/day	74 (4.9%)	11 (5.3%)	1.08 (0.57–2.10)	0.99 (0.38–2.56)	40 (3.9%)	0.78 (0.53–1.16)	0.65 (0.36–1.17)	33 (4.7%)	5 (5.0%)	1.07 (0.41–2.80)	1.78 (0.49–6.41)	21 (4.0%)	0.86 (0.49–1.51)	0.96 (0.42–2.19)	41 (5.2%)	6 (5.7%)	1.10 (0.46–2.66)	0.60 (0.14–2.59)	19 (3.8%)	0.72 (0.41–1.25)	0.45 (0.19–1.07)
**Cholesterol**																					
<300 mg/day	60 (4.0%)	6 (2.9%)	Ref.	Ref.	54 (5.3%)	Ref.	Ref.	24 (3.4%)	3 (3.0%)	Ref.	Ref.	21 (4.0%)	Ref.	Ref.	36 (4.6%)	3 (2.9%)	Ref.	Ref.	33 (6.6%)	Ref.	Ref.
>300 mg/day	1436 (96.0%)	200 (97.1%)	1.39 (0.59–3.27)	1.47 (0.52–4.18)	968 (94.7%)	0.75 (0.51–1.09)	0.69 (0.45–1.07)	685 (96.6%)	98 (97.0%)	1.15 (0.34–3.87)	2.37 (0.31–18.11)	499 (96.0%)	0.83 (0.46–1.51)	0.88 (0.44–1.74)	751 (95.4%)	102 (97.1%)	1.63 (0.49–5.39)	1.16 (0.34–3.95)	469 (93.4%)	0.68 (0.42–1.11)	0.59 (0.34–1.04)
**Water**																					
>1600 mL/day	188 (12.6%)	20 (9.7%)	Ref.	Ref.	136 (13.3%)	Ref.	Ref.	91 (12.8%)	11 (10.9%)	Ref.	Ref.	75 (14.4%)	Ref.	Ref.	97 (12.3%)	9 (8.6%)	Ref.	Ref.	61 (12.2%)	Ref.	Ref.
<1600 mL/day	1308 (87.4%)	186 (90.3%)	1.34 (0.82–2.17)	1.42 (0.72–2.81)	886 (86.7%)	0.94 (0.74–1.19)	0.91 (0.66–1.26)	618 (87.2%)	90 (89.1%)	1.21 (0.62–2.34)	1.28 (0.49–3.40)	445 (85.6%)	0.87 (0.63–1.21)	0.98 (0.62–1.55)	690 (87.7%)	96 (91.4%)	1.50 (0.73–3.07)	1.52 (0.58–3.96)	441 (87.8%)	1.02 (0.72–1.43)	0.86 (0.55–1.34)

^1^ Saturated fatty acids. ^2^ Monounsaturated fatty acids. ^3^ Polyunsaturated fatty acids. * Given that no children met the recommended intakes, medians were used as the cutoff points for calculation of the OR.

## Data Availability

Data described in the manuscript will not be made available because of ethical privacy concerns.
